# Novel pH-Responsive PSS-Loaded Chitosan Matrix Nanoparticles Ameliorate Pressure Overload-Induced Cardiac Hypertrophy

**DOI:** 10.3390/md23090365

**Published:** 2025-09-19

**Authors:** Meijie Xu, Zhen Fan, Dingfu Wang, Dan Li, Haimiao Zou, Yiting Xue, Shixin Wang, Chunxia Li

**Affiliations:** 1 Key Laboratory of Marine Drugs of the Ministry of Education, Shandong Key Laboratory of Glycoscience and Glycotherapeutics, School of Medicine and Pharmacy, Ocean University of China, Qingdao 266003, China; xumeijie@stu.ouc.edu.cn (M.X.); fanzhen009@126.com (Z.F.); wangdingfu@stu.ouc.edu.cn (D.W.); haimiaozou@163.com (H.Z.); 2 Laboratory for Marine Drugs and Bioproducts, Qingdao Marine Science and Technology Center, Qingdao 266237, China; 3 School of Biology and Food Engineering, Suzhou University of Technology, Suzhou 215000, China; 4 Laboratory of Marine Glycodrugs Research and Development, Marine Biomedical Research Institute of Qingdao, Qingdao 266237, China

**Keywords:** propylene glycol alginate sulfate sodium, polyelectrolyte complex, glycocholic acid, trimethyl chitosan, myocardial hypertrophy

## Abstract

Cardiac hypertrophy is a critical contributor to cardiac dysfunction and the development of heart failure, yet effective therapeutic strategies remain limited. Propylene glycol alginate sulfate sodium (PSS) is a marine sulfated polysaccharide drug used in the treatment of cardiovascular diseases and has shown cardiac function benefits. Here, we designed a pH-responsive PSS-loaded nanoparticle drug delivery system. It was self-assembled by negatively charged PSS with positively charged trimethyl chitosan glycocholic acid (TMC-GA) via electrostatic interaction, and further stabilized the nanoparticles with Hydroxypropyl methylcellulose phthalate (HP55) excipients. The prepared TMC-GA/HP55@PSS nanoparticles were spherical, with a mean particle size of 361.5 ± 1.26 nm, zeta potential of −30.3 ± 0.9 mV, and encapsulation efficiency of 92.52 ± 2.4%. In vitro release study demonstrated the pH-responsive property of TMC-GA/HP55@PSS under intestinal conditions and facilitated nanoparticles absorption in the intestinal epithelium. In vitro experiments confirmed the biocompatibility of PSS and its ability to improve myocardial cell hypertrophy. In vivo, both PSS and its nanoparticles significantly ameliorated pressure overload–induced cardiac hypertrophy in mice, with TMC-GA/HP55@PSS exhibiting better cardioprotective efficacy. This study is the first to integrate pH-responsiveness and bile acid transport-mediated uptake into PSS nanocarrier systems. The findings provide valuable data and enlightenment for designing novel formulations and expanding the clinical applications of PSS.

## 1. Introduction

Propylene glycol alginate sodium sulfate (PSS) is a marine sulfated polysaccharide drug consisting of repeating units of polyguluronic acid (PG) and polymannuronic acid (PM) [1]. PSS is a polyanionic polysaccharide with a heparin-like chemical structure and physiological activity. It has been used as an anticoagulant in clinical practice for around 40 years [2]. Recently, it has been found that PSS exhibits its anti-hepatic fibrosis activity by participating in the TGF-β/Smad2/3 and JAK/STAT signaling pathways [3]. Mao et al. found that PSS-NP could improve myocardial morphology, cardiac function, and coronary microcirculation in rat models, suggesting a promising cardioprotective role [4]. PSS is widely used in the treatment of cardiovascular diseases due to its anticoagulant, lipid-lowering, anti-fibrotic, anti-inflammatory, and coronary microcirculation-improving properties [5].

Pathologic cardiac hypertrophy is usually triggered by hypertension, myocardial infarction, and aortic stenosis [6]. Cardiac hypertrophy is an adaptive response of the heart to stress overload or neurohumoral stimulation, with its molecular mechanisms involving multiple interconnected signaling pathways [7]. These include the G protein-coupled receptor (GPCR) pathway [8], calcineurin-NFAT pathway [9], PI3K/Akt/mTOR pathway [10], inflammatory signaling pathways [11], and stress-activated pathways (e.g., JAK/STAT) [12]. This network coordinates processes including cardiomyocyte size regulation, protein synthesis, re-expression of embryonic genes, and fibrosis. Although numerous studies have reported the pathophysiological mechanisms of pathological myocardial hypertrophy progressing to heart failure, there still lacks effective therapeutic drugs [13]. Based on the therapeutic effects of PSS in cardiovascular diseases, we aim to develop a novel PSS nanoparticle formulation to enhance its oral bioavailability and ameliorate the pathological characteristics of myocardial hypertrophy.

PSS is a highly hydrophilic polysaccharide sulfate ester. Currently, dosage forms of PSS used in clinical practice are tablets and injections. Although injections are highly effective, they can cause adverse reactions [14]. Tablets are convenient to take but have the disadvantage of low oral bioavailability due to their high molecular weight [15]. Therefore, many PSS formulations have been developed, such as PSS liposomes and PSS soft capsules [16]. However, their drug encapsulation rates are all around 70%, and they have a large number of excipients and complex preparation processes. Therefore, the development of novel delivery strategies to overcome these challenges is significantly important.

Polyelectrolyte complexes (PECs) are formed when oppositely charged natural or synthetic polymers combine through electrostatic interactions [17]. For instance, positively charged chitosan or its derivatives, along with negatively charged glycosaminoglycans, self-assemble through electrostatic interactions to form stable nanoparticles [18,19]. These complexes can be formed without chemical cross-linking agents and are biocompatible, degradable, and structurally tunable [20]. They are gradually used in drug delivery systems.

The oral administration of drug delivery systems requires overcoming the intestinal absorption barriers. It has been reported that numerous receptors and transporters are distributed on intestinal epithelial cells, such as folate receptor, glucose transporter, and bile acid transporter [21]. The efficiency of bile acid enterohepatic circulation is approximately 95%, mainly relying on the Apical sodium-dependent bile acid transporter (ASBT) on the top side of ileal intestinal cells [22]. Glycocholic acid (GCA), a derivative of bile acids, can be absorbed and transported more efficiently by ASBT in the small intestinal membrane [23]. Therefore, surface modification of drug carriers using GCA can greatly facilitate the transportation and absorption of drugs in the small intestine.

Hydroxypropyl methylcellulose phthalate (HPMCP) is widely used as a good enteric coating agent to protect drug components from damage by gastric acid [24]. HP55 is a special type of HPMCP with solubility at pH above pH 5.5, which improves the stability of drugs in the stomach [25]. HP55/poly(N-butyl cyanoacrylate) (PBCA) nanoparticles encapsulating a Helicobacter pylori subunit vaccine were prepared by Hai Liu et al. [26]. This acid- and protein-hydrolysis-resistant carrier was used to prevent *H. pylori* infections in mice via oral administration.

**Scheme 1:**
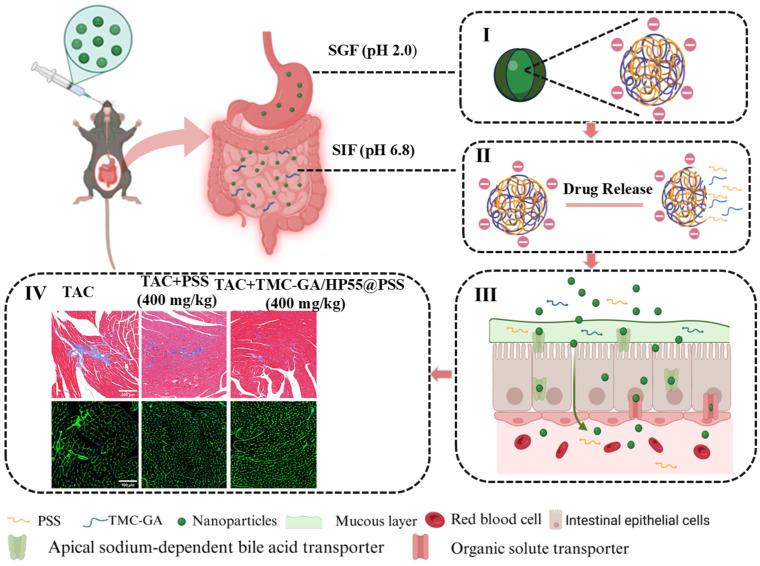
The oral absorption process of PSS and TMC-GA/HP55@PSS. (**I**) The nanoparticles maintained their structural integrity in the simulated gastric fluid. (**II**) Nanoparticles undergo slow drug release in the simulated intestinal fluid. (**III**) Transport of nanoparticles in the intestinal tract. (**IV**) The improvement of PSS and nanoparticles on myocardial hypertrophy.

Therefore, to improve the intestinal absorption of PSS and evaluate its effect on myocardial hypertrophy, we designed a novel pH-responsive PSS-loaded chitosan matrix nanoparticle (Scheme 1). The PECs were formed via electrostatic interactions between trimethyl chitosan glycocholic acid derivatives (TMC-GA) and PSS under acidic conditions. And the enteric material HP55 was added to the prescription to protect PSS from being damaged in the stomach environment. When the PSS-loaded PEC nanoparticles arrive in the intestine, the alkaline environment will promote the release of PSS. In addition, the synergistic effect of chitosan’s promoting absorption and the bile acid transporter on intestinal epithelial cells will accelerate the intestinal absorption of nanoparticles. These integrated strategies will improve the oral bioavailability of PSS, thereby enhancing its efficacy in ameliorating myocardial hypertrophy.

## 2. Results and Discussion

### 2.1. Preparation and Characterization of PSS Nanoparticles

**Figure 1:**
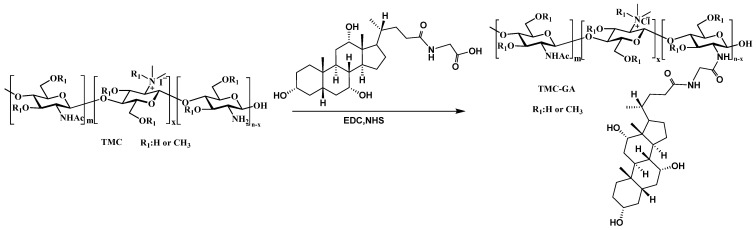
Synthesis route of trimethyl chitosan glycocholic acid derivative (TMC-GA).

The novel pH-responsive PSS polyelectrolyte complex nanoparticles were prepared by the ion cross-linking method [27]. Firstly, the trimethyl chitosan glycocholic acid derivative (TMC-GA, Figure 1) was synthesized through amide condensation reaction between trimethyl chitosan and glycocholic acid [28], followed by dialysis, concentration, and freeze-drying to yield a white powder. The degree of glycocholic acid substitution in TMC-GA was 12%.

**Figure 2:**
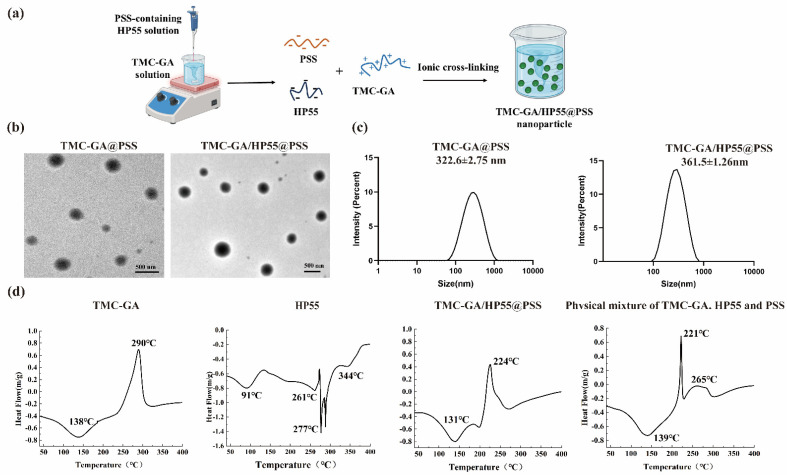
(**a**) Preparation of PSS nanoparticles. (**b**) Transmission electron micrographs of PSS nanoparticles and their particle size distribution. (**c**) Statistical graph of particle size of PSS nanoparticles by Malvern laser particle sizer. (**d**) Differential scanning calorimetry analysis of PSS nanoparticles.

After obtaining glycocholic acid-modified chitosan, the positively charged TMC-GA and the polyanionic polysaccharide PSS formed nanoparticles through electrostatic interactions with or without the enteric material HP55, as shown in Figure 2a. Two kinds of PSS nanoparticles, TMC-GA@PSS and TMC-GA/HP55@PSS, were prepared through single-factor experiments optimization, and their morphology, particle size, encapsulation efficiency, and zeta potential were systematically evaluated to confirm successful nanoparticle construction. 

The drug encapsulation rate of TMC-GA/HP55@PSS was 92.52 ± 2.4% and the drug loading was 16.3 ± 0.34% by the carbazole sulfate method [29]. The morphology of TMC-GA@PSS and TMC-GA/HP55@PSS was spherical or spheroidal structure, observed by transmission electron microscopy (Figure 2b), with the particle sizes 267.99 ± 59.48 nm and 337.56 ± 59.90 nm, respectively (Figure S3). Then, using a Malvern laser particle sizer (Figure 2c), the particle size of TMC-GA@PSS and TMC-GA/HP55@PSS were 322.6 ± 2.75 nm and 361.5 ± 1.26 nm, respectively. The corresponding polydispersity index (PDI) values were 0.24 ± 0.02 and 0.12 ± 0.08, respectively. The particle size of nanoparticles determined by Malvern is larger than that determined by TEM, because Malvern measures the hydrated diameter of nanoparticles, while TEM directly images particles in a dry state. Compared with TMC-GA@PSS, the increased particle size of TMC-GA/HP55@PSS was mainly due to the electrostatic interaction of HP55 adsorbing on the surface of TMC-GA molecules. Furthermore, the Zeta potential of TMC-GA@PSS was +30.5 ± 1.2 mV, while that of TMC-GA/HP55@PSS nanoparticles was −30.3 ± 0.9 mV. The Zeta potential of the PECs changed from positive to negative, mainly due to the negatively charged HP55 adsorbing on the positively charged surface of TMC-GA. 

Furthermore, the structural changes of nanoparticles were verified by differential scanning calorimetry analysis (Figure 2d). In the DSC spectrum of TMC-GA/HP55@PSS nanoparticles, the endothermic peak at 131 °C was close to the main component of TMC-GA, and the exothermic peak at 224 °C might be the energy released after the intermolecular interaction force between the excipient and the main drug was disrupted. Moreover, the nanoparticles of TMC-GA/HP55@PSS lacked the exothermic peak at 265 °C found in physical mixtures. This also indicated that the nanoparticles were not a simple physical mixture of the excipients and PSS, but were combined through non-covalent interactions.

### 2.2. Drug Release Study of TMC-GA/HP55@PSS Nanoparticles

**Figure 3:**
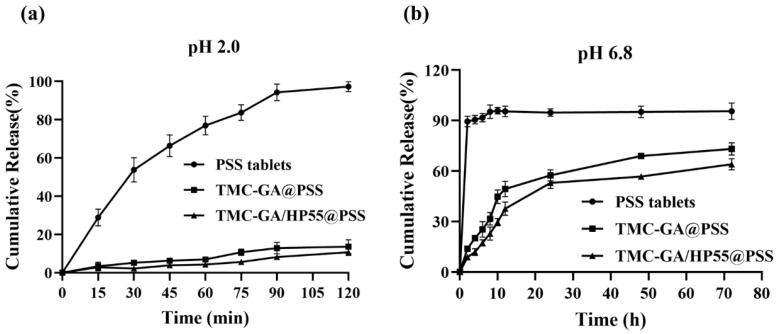
Cumulative drug release of PSS tablets and PSS polyelectrolyte complex nanoparticles in artificial gastric fluid (SGF) and artificial intestinal fluid (SIF) at 37 °C (*n* = 3). (**a**) Cumulative drug release in SGF (pH 2.0). (**b**) Cumulative drug release in SIF (pH 6.8).

To determine the pH responsiveness of HP55, we compared the release profiles of PSS tablets, TMC-GA@PSS, and TMC-GA/HP55@PSS in artificial gastric fluid (SGF, pH 2.0) and artificial intestinal fluid (SIF, pH 6.8). Figure 3a showed that both TMC-GA@PSS and TMC-GA/HP55@PSS could maintain their structural stability at pH 2.0 with a cumulative drug release of 13.59% and 10.74% after 2 h, respectively. However, the PSS tablets released the drug rapidly in simulated gastric fluid with a cumulative drug release of 97.32% after 2 h. It suggested that PSS polyelectrolyte complexes can protect PSS from damage in the gastric acidic environment. Owing to protonation of TMC-GA under acidic conditions, its interaction with PSS was more stabilized. In addition, the protective effect of the enteric material HP55 resulted in a reduced cumulative drug release from TMC-GA/HP55@PSS. 

As shown in Figure 3b, the cumulative drug release of PSS tablets in simulated intestinal fluid after 4 h was 90.53%, and the cumulative drug release of TMC-GA@PSS and TMC-GA/HP55@PSS after 12 h was 49.37% and 37.67%, respectively. The results indicated that the gradual dissolution of HP55 in the intestinal environment enables the nanoparticles to achieve pH responsiveness, thereby protecting PSS from degradation in the stomach environment. During intestinal absorption, the deprotonation of TMC-GA molecules resulted in a decrease in intermolecular interactions, leading to the slow release of PSS.

### 2.3. Biosafety Evaluation of TMC-GA/HP55@PSS Nanoparticles

**Figure 4:**
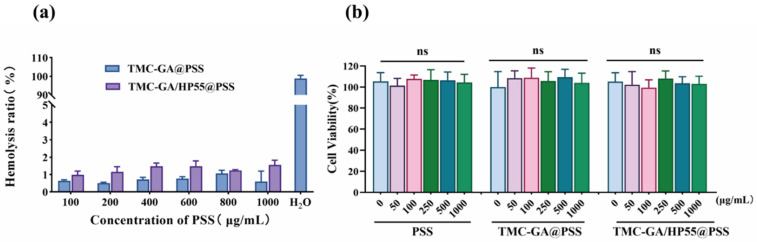
In vitro safety evaluation of PSS and its nanoparticles. (**a**) Hemolysis rate of different doses of PSS nanoparticles. Statistical data are expressed as mean ± SD (*n* = 3). (**b**) Cell survival rate of different doses of PSS and its nanoparticles. Data are presented as the means ± SD (*n* = 6), ns indicates no significant difference.

The hemolysis rate and cytotoxicity of different doses of PSS and its nanoparticles were assessed to verify the biosafety of nanoparticles. The hemolysis rates of TMC-GA@PSS and TMC-GA/HP55@PSS were below 5% in the PSS concentration range of 0 to 1000 μg/mL (Figure 4a), which was consistent with the literature reports [30]. In addition, the viability of H9C2 cells after co-incubation with PSS and its two formulations for 24 h was assessed using the CCK8 assay. Cell viability exceeded 90% at all tested concentrations of PSS and its formulations (Figure 4b). These results indicate that PSS and its nanoparticles have good biosafety.

### 2.4. In Vitro Efficacy of PSS Against Cardiomyocyte Hypertrophy

To investigate the therapeutic potential of PSS on cardiac hypertrophy, a H9C2 cell hypertrophy model was constructed using different concentrations of Ang II [31]. Subsequently, hypertrophic gene expression was quantified using real-time quantitative PCR (qRT-PCR), including atrial natriuretic peptide (ANP), β-myosin heavy chain (β-MHC), and brain natriuretic peptide (BNP). After the hypertrophy model was successfully established, the cells were treated with varying concentrations of PSS, with telmisartan used as a positive control. The cell morphology in each group was observed using a laser confocal microscope, and the expression levels of ANP protein were assessed by Western blot analysis.

**Figure 5:**
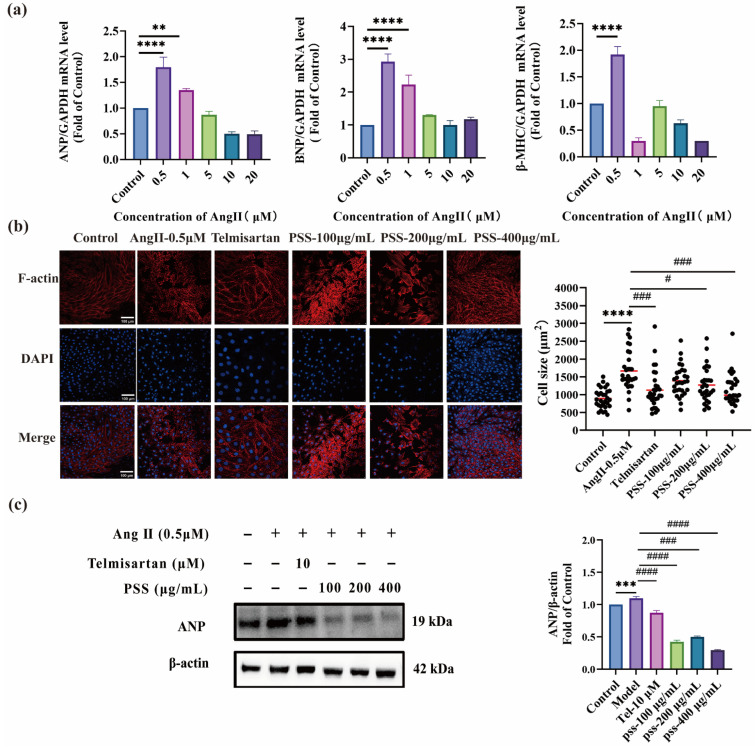
In vitro evaluation of the efficacy of PSS on ameliorating hypertrophy. (**a**) Induction of H9C2 Cardiac Hypertrophy with Varying Concentrations of Ang II. (**b**) The effect of PSS on the cell area of H9C2 Cardiac Hypertrophy, scale bar: 100 μm. (**c**) Effect of PSS on ANP Expression in H9C2 Cardiac Hypertrophy. Data are expressed as the means ± SD (*n* = 3). Significance: ** *p* < 0.01, *** *p* < 0.001, **** *p* < 0.0001, versus Control group; # *p* < 0.05, ### *p* < 0.001, #### *p* < 0.0001, versus model group.

As shown in Figure 5a, in the model group, the average relative expression levels of ANP, BNP, and β-MHC mRNA in H9C2 cells were 1.796, 2.928, and 1.919, respectively, which increased by 1.8-fold, 2.9-fold, and 1.9-fold, respectively, compared with the Control group. The results of the qRT-PCR assay showed that 0.5 µM Ang II could be used to induce the H9C2 cell hypertrophy model. As shown in Figure 5b, cell area increased by 1.6-fold in the model group compared to the control group. In contrast, co-incubation with Telmisartan (10 μM) and PSS (400 μg/mL) induced a significant decrease in cell area, comparable to that of the control group, indicating that PSS has a protective effect against cardiac hypertrophy.

The ANP protein expression was further analyzed by Western blot (Figure 5c). In the model group, ANP expression was significantly upregulated, whereas in the Telmisartan (10 μM) group and PSS-treated group, ANP expression was significantly reduced. Among them, the expression level of ANP protein in the Model group was four times that of the PSS (400 μg/mL) group. These findings suggested that PSS effectively ameliorates myocardial hypertrophy, with results aligning with those of the cell area assay. 

### 2.5. In Vivo PSS and Its Nanoparticles Ameliorated Cardiac Hypertrophy

To evaluate the effect of PSS and its nanoparticles on cardiac hypertrophy in vivo, we established the myocardial hypertrophy model induced by pressure overload through TAC surgery [32]. Over the 4-week period, the mice behaved normally and no significant change in body weight (Figure S1), suggesting that the drug formulation was safe in vivo. After four weeks of intragastric administration, the effects of PSS and TMC-GA/HP55@PSS on cardiac function and cardiac index were assessed, with telmisartan as the positive control. In addition, Masson’s trichrome and wheat germ agglutinin (WGA) staining were used to evaluate changes in myocardial fibrosis and myocardial cell area in mice with myocardial hypertrophy, further elucidating the therapeutic effects of PSS and its formulations on the pathological features of myocardial hypertrophy.

#### 2.5.1. Echocardiography Analysis

Myocardial hypertrophy is associated with cardiac dysfunction, characterized by a reduction in left ventricular short-axis shortening fraction (LVFS) or left ventricular ejection fraction (LVEF). Concurrently, a relative increase in the thickness of the interventricular septum (IVSD) and in the left ventricular internal diameter during systole and diastole (LVIDs, LVIDd) was observed [33]. Therefore, the improvement of contractile and diastolic functions in mice with myocardial hypertrophy is a key parameter for evaluating the therapeutic efficacy of drugs.

**Figure 6:**
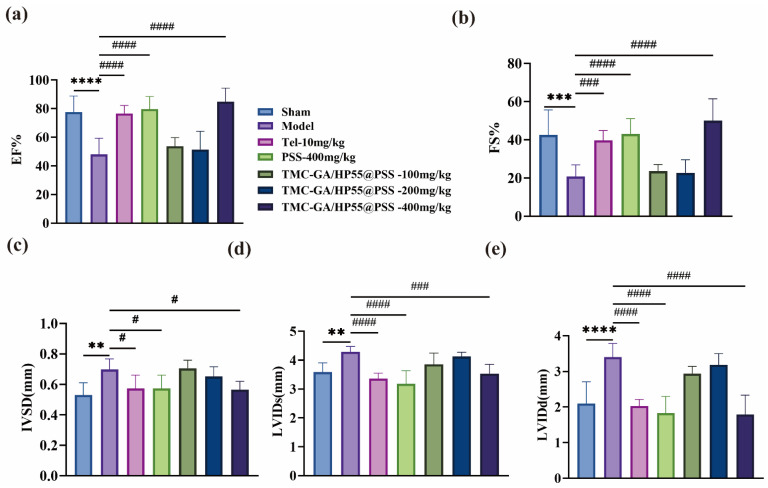
Echocardiographic parameters of different groups (**a**) EF, (**b**) FS, (**c**) IVSD, (**d**) LVIDs, (**e**) LVIDd. Data are expressed as the means ± SD (*n* = 6). Significance: **** *p* < 0.0001, *** *p* < 0.001, ** *p* < 0.01, compared with Sham group; # *p* < 0.05, ### *p* < 0.001, #### *p* < 0.0001, compared with model group.

At the end of the 4-week treatment, echocardiography was performed to assess the effects of PSS and its nanoparticles on cardiac function (Figure 6). Compared with the model group, treatment with Telmisartan (10 mg/kg/day), PSS (400 mg/kg/day), and TMC-GA/HP55@PSS (400 mg/kg/day) led to a marked improvement in cardiac function. Specifically, the ejection fraction (EF) increased from 50% to 80%, the shortening fraction (FS) increased from 20% to 50% (Figure 6a,b), and IVSD, LVIDs, and LVIDd significantly decreased (Figure 6c–e). These effects were comparable to those observed in the Sham group. These results suggested that treatment with PSS (400 mg/kg/day) and TMC-GA/HP55@PSS (400 mg/kg/day) effectively facilitated the recovery of cardiac function.

#### 2.5.2. Histopathologic Analysis of Myocardial Tissue

**Figure 7:**
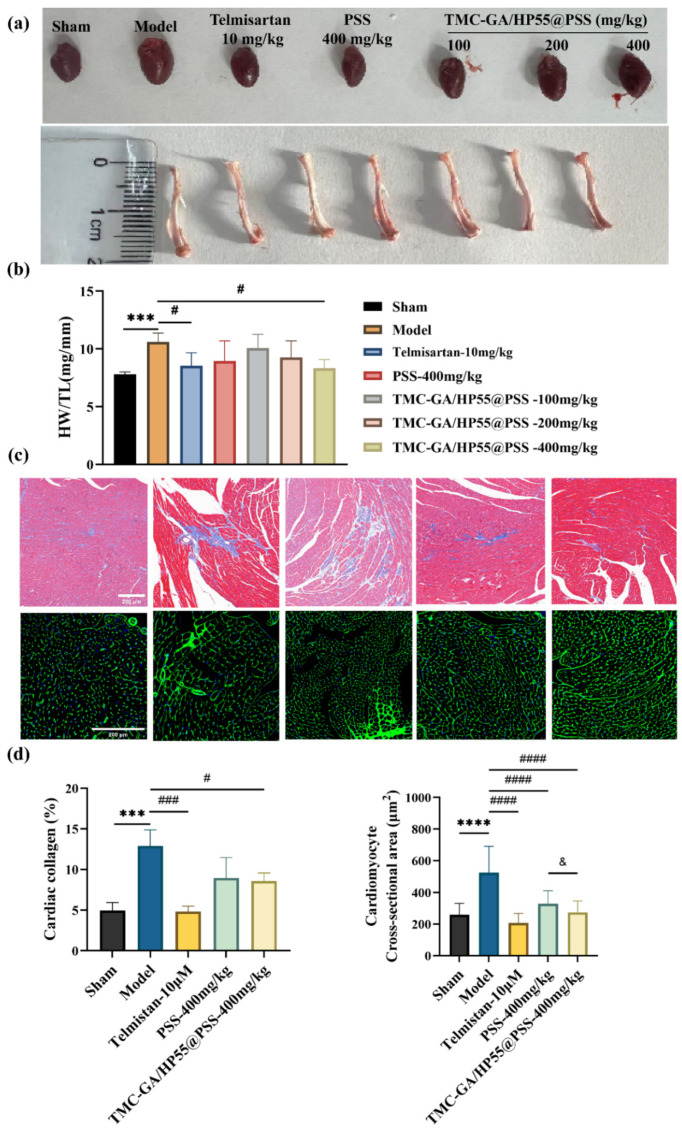
(**a**) Representative morphological images of heart tissue and left tibia. (**b**) Ratios of heart weight to left tibia length. (**c**) Masson staining and WGA staining. Scale bar: 200 µm. (**d**) Statistical results of cardiac collagen and cardiomyocyte cross-sectional area. Data are expressed as the means ± SD (*n* = 6). Significance: *** *p* < 0.001, **** *p* < 0.001, compared with Sham group; # *p* < 0.05, ### *p* < 0.001, #### *p* < 0.0001, compared with model group; & *p* < 0.05, compared TMC-GA/HP55@PSS with PSS group.

The heart weight to left tibia length ratio (HW/TL, mg/mm) was calculated and used as an indicator of cardiac hypertrophy, reflecting the extent of myocardial hypertrophy [34]. After four weeks of gavage administration, the hearts and left tibias of the mice were collected (Figure 7a). The heart weight to left tibia length ratio (HW/TL, mg/mm) (Figure 7b) revealed a significant increase in heart size in the Model group compared with the sham group. In contrast, heart size in the Telmisartan-10 mg/kg/day and TMC-GA/HP55@PSS-400 mg/kg/day treatment groups approached that of healthy mice, indicating that PSS could alleviate the pathological features of cardiac hypertrophy. The results of the animal experiments were also consistent with those at the cellular level, further indicating that PSS had the effect of improving myocardial hypertrophy.

HE staining results showed myocardial fibers arranged neatly with no obvious inflammatory cell infiltration (Figure S2), suggesting the nanoparticles had good safety [35]. Since the collagen deposition and the myocardial cell cross-sectional area can directly reflect the development of pathological myocardial hypertrophy [36]. Therefore, we performed Masson trichrome staining and WGA staining of myocardial tissues in each group (Figure 7c). The Masson’s trichrome staining results revealed that treatment with telmisartan (10 mg/kg/day) and TMC-GA/HP55@PSS (400 mg/kg/day) significantly reduced collagen deposition in the myocardial tissue, compared to the Model group. After staining with wheat germ lectin (WGA), the cross-sectional area results of fifty cardiomyocytes were randomly counted (Figure 7d). The mean cell areas of model, telmisartan (10 mg/kg/day), PSS (400 mg/kg/day), and TMC-GA/HP55@PSS (400 mg/kg/day) treatment groups were 259.19, 524.50, 206.85, 328.96, and 274.29 μm^2^, respectively. Compared with the model group, the cross-sectional area of cardiomyocytes in the telmisartan (10 mg/kg/day), PSS (400 mg/kg/day), and TMC-GA/HP55@PSS (400 mg/kg/day) treatment groups was reduced by 2.5-fold, 1.5-fold, and 2.0-fold, respectively. These findings indicated that the TMC-GA/HP55@PSS (400 mg/kg/day) treatment group exhibited a more pronounced improvement.

Our results showed that PSS and its nanoparticles mitigate pressure overload-induced cardiac hypertrophy in TAC mice. Moreover, the therapeutic effect of TMC-GA/HP55@PSS is better than that of free PSS, further indicating that TMC-GA/HP55@PSS enhanced the oral absorption of PSS. 

PSS is an acidic anionic polysaccharide, and the oral absorption of such biomacromolecules is influenced by various factors [37]. Leveraging the anionic nature of PSS and the cationic properties of trimethyl chitosan glycocholic acid derivative, PEC nanoparticles were prepared. Furthermore, we added the enteric material HP55 to the formulation prescription, which enables the pH-responsivity of the nanoparticles and protects the formulations from being damaged by the stomach environment. During intestinal absorption, the TMC-GA/HP55@PSS nanoparticles had a significant sustained-release effect. Thus, the nanoparticles could utilize the absorption-enhancing effects of trimethyl chitosan and bile acid transporters on the intestinal cell surface, thereby facilitating intestinal transport and improving the oral bioavailability of PSS.

Recent studies have shown that the component derivative PGGS had a significant inhibitory effect on the extracellular matrix by atrial fibroblasts. PGGS exhibited anti-atrial fibrosis activity by specifically inhibiting the TGF-β1/Smad2/3 signaling pathway [38]. However, acute toxicity had been observed in animal experiments, and the cause remains unclear at present. The pathological myocardial hypertrophy is usually associated with myocardial vascular damage, extracellular matrix deposition, and myocardial fibrosis [39]. As a marketed marine drug, PSS is mainly used for anticoagulant, antithrombotic, and hypolipidemic [2]. Therefore, we used Ang II to induce cardiac hypertrophy in H9C2 cells to evaluate the potential effect of PSS. The results demonstrated that PSS directly inhibited expression of fetal isoforms of ANP, BNP, and β-MHC in cardiomyocytes, suggesting a direct anti-hypertrophic effect. Furthermore, we established a TAC mouse model [40]. After 4-week oral gavage administration, we found that significantly reduced levels of RAAS markers were observed following PSS and its formulations treatment, demonstrating that PSS might modulate key humoral pathways implicated in myocardial hypertrophy, thereby indirectly alleviating pathological cardiac stress. These results indicated that PSS might exert synergistic cardioprotective effects by targeting both intrinsic hypertrophic pathways in cardiomyocytes and extrinsic pathological neurohumoral stimuli.

Taken together, the anticoagulant effect of PSS primarily results from its inhibition of thrombin activity. According to literature reports, thrombin, as a multifunctional serine protease, may play a role in cardiac remodeling by promoting inflammatory responses and tissue necrosis [41]. We speculate that this might be the potential mechanism by which PSS improves the pathological features of myocardial hypertrophy. Nevertheless, several limitations remain. The precise molecular mechanisms by which PSS attenuates pathological remodeling, such as its effects on inflammatory signaling, oxidative stress, and fibrosis-related pathways, need further elucidation. Furthermore, the transport mechanisms of both nanoparticles will require detailed investigation in future studies to directly validate the synergistic effect of HP55 and ABST on nanoparticle absorption. In addition, future investigations in clinical settings are essential to validate the translational potential of this approach.

## 3. Materials and Methods

### 3.1. Materials and Reagents

Propylene glycol alginate sodium sulfate (PSS-231232002, Mw 24 kDa, S% 12.79%, M/G 2.91) obtained from Henan Tianfang Pharmaceutical Co., Ltd. (Zhumadian, Henan, China). Trimethyl chitosan with 40% trimethylation and a molecular weight of 16.57 kDa was prepared in our laboratory. Glycocholic acid was purchased from Aladdin Biochemical Technology Co., Ltd., (Shanghai, China). Hydroxypropyl methylcellulose phthalate was purchased from Hubei Xinyuhong Biomedical Technology Co., Ltd., (Wuhan, Hubei, China). Sodium hydroxide, hydrochloric acid, 1-(3-Dimethylaminopropyl)-3-ethylcarbodiimide hydrochloride (EDCI), carbazole, sulfuric acid, borax, and N-hydroxysuccinimide (NHS) were all purchased from Shanghai Sinopharm Group Chemical Reagent Co., Ltd., (Shanghai, China). Angiotensin II was purchased from MedChemExpress Biotech Company (Shanghai, China). qPCR reagents for real-time reverse transcriptase analysis were supplied by Nanjing Novozyme Biotechnology Co., Ltd., (Nanjing, China). 

### 3.2. Synthesis and Characterization of TMC-GA

TMC-GA was synthesized and purified using a method previously reported [42]. It was synthesized by one-step coupling of trimethyl chitosan and glycocholic acid via EDCI/NHS reaction. Firstly, 0.25 g glycocholic acid was dissolved in 50 mL of CH3OH solution, followed by the addition of 288 mg of EDCI and 173 mg of NHS, and the mixture was stirred for 30 min. Then, 0.5 g TMC was dissolved in 50 mL of distilled water, which was gradually added to the glycocholic acid solution. The reaction was carried out for 24 h at 25 °C in the absence of light. After the reaction, the reaction solution was dialyzed sequentially with a mixture of methanol/water (2:1, vol:vol) and water for 2 days, respectively. The solution was then concentrated and lyophilized to obtain TMC-GA.

Then, 30 mg of the TMC-GA derivatives were dissolved in 0.5 mL of D_2_O, and were analyzed by ^1^H-NMR (Bruker Avance NEO 400 MHz, Bruker Corporation, Berlin, Germany) at 25 °C. The GA grafting degree of TMC-GA was calculated from the ^1^H-NMR spectra reported previously [43].

### 3.3. Preparation and Characterization of PSS Nanoparticles

First, 50 mg of the TMC-GA compound was dissolved in 20 mL of distilled water to obtain a 2.5 mg/mL solution. The pH was then adjusted to about 3 using 1 mol/L HCl solution. Then, 20 mg of HP55 and 20 mg of PSS were dissolved in 3 mL of 0.1 mol/L NaOH solution, and the final concentration of the mixed solution of HP55 and PSS was adjusted to 2.0 mg/mL using 7 mL of distilled water. The pH of the solution was adjusted to approximately 5.5 with 1 mol/L HCl solution. Finally, the PSS polyelectrolyte complexes (TMC-GA/HP55@PSS) were formed by slowly adding the HP55 and PSS mixed solution to three times the volume of the TMC-GA solution, and the pH was adjusted to 2 using 1 mol/L HCl. The PSS polyelectrolyte complexes were sonicated at 120 W for 5 min, with a work-up of 2 s and an interval of 3 s. The PSS polyelectrolyte complex nanoparticles without HP55 (TMC-GA @PSS) were prepared using the same method.

The morphology of TMC-GA@PSS and TMC-GA/HP55@PSS nanoparticles was observed by transmission electron microscopy (TEM, JEM-2100, JEOL Ltd., Tokyo, Japan). The particle size, particle size distribution, and Zeta potential (10 mg·mL^−1^) of TMC-GA@PSS and TMC-GA/HP55@PSS nanoparticles were characterized by using a laser particle size analyzer (Zetasizer Nano ZS, Malvern, UK). 

The differential scanning calorimetry (DSC) curves of HP55, TMC-GA, TMC-GA/HP55@PSS, and the physical mixtures of PSS, TMC-GA, and HP55 were determined. The crucible temperature was increased from 20 to 400 °C at a rate of 10 °C/min^−1^, and the flow rate of nitrogen was 50 mL/min.

### 3.4. Encapsulation Efficiency of Nanoparticles

The encapsulation efficiency of PSS was determined by the carbazole sulfate method [29].

The free drug was removed by ultrafiltration. Briefly, 4 mL of the nanoparticle suspension was processed with a 100 kDa ultrafiltration tube. The tubes were subsequently washed with distilled water three times to disperse the nanoparticles, which were then destroyed using a 2 mol/L NaOH solution. Subsequently, the solution was centrifuged at 13,000 rpm for 10 min, and the supernatant was collected. The PSS content was determined using the carbazole sulfate method. The encapsulation efficiency (EE) and drug loading (DL) of TMC-GA@PSS and TMC-GA/HP55@PSS were calculated according to the following equations.

EE (%) = Content of PSS in nanoparticles/Total amount of PSS added × 100% (1)

DL (%) = Content of PSS in nanoparticles/Total dry weight of nanoparticles × 100%(2)

### 3.5. Release of PSS from the Nanoparticles

The in vitro release of TMC-GA@PSS and TMC-GA/HP55@PSS nanoparticles was determined by the carbazole sulfate method [30]. 

Firstly, 2 milliliters of TMC-GA@PSS and TMC-GA/HP55@PSS nanoparticles were separately added to sample bottles containing 5 milliliters of simulated gastric fluid (with a pH value of 2) using a pipette. Then, the sample bottles were placed on an oscillator set at 100 rpm/min and 37 °C to measure the cumulative drug release. The samples were collected at 15, 30, 45, 60, 75, 90, and 120 min, respectively, and processed by using an ultrafiltration tube of 100 kDa. The ultrafiltrate solution was discarded, and the ultrafiltration tube was washed with distilled water 3 times to disperse the nanoparticles. The nanoparticles were disrupted by 2 mol/L NaOH solution, centrifuged at 13,000 rpm for 10 min, and the supernatant was collected for PSS quantification.

Then, 2 mL of TMC-GA@PSS and TMC-GA/HP55@PSS were added to 5 mL of simulated intestinal fluid (pH 6.8), respectively. Nanoparticle solutions were taken at 2, 4, 6, 8, 10, 12, 24, 48, and 72 h, respectively. The samples were processed by using an ultrafiltration tube of 100 kDa, and the ultrafiltrate was collected for PSS quantification.

### 3.6. Biosafety Evaluations of Nanoparticles

#### 3.6.1. In Vitro Cytotoxicity of Nanoparticles

The cytotoxicity of the nanoparticles was assessed using the CCK-8 assay in H9C2 cells. The cells were seeded at a density of 8 × 10^3^ cells/well in 96-well plates and cultured for 24 h at 37 °C in a 5% CO_2_ incubator. The cells were then treated with TMC-GA@PSS and TMC-GA/HP55@PSS at designated concentrations for a further 24 h. The medium was subsequently removed and replaced with 10 μL of CCK-8 reagent, after which the cells were cultured for one hour at 37 °C. The optical density (OD) values were then measured at 450 nm using a Spark 10 M microplate reader (Tecan Trading AG, Männedorf, Switzerland). Each sample was analyzed in six replicates. Cell viability was calculated according to Equation (3).

Cell viability (%) = (A_sample_ − A_blank_)/(A_control_ − A_blank_) × 100% (3)

where A_sample_ refers to the OD values of cells treated with samples, A_control_ refers to the OD values of cells treated without samples, and A_blank_ is the OD value of the cell culture medium.

#### 3.6.2. Hemolytic Test of Nanoparticles

In brief, rat red blood cells (RBCs, 4% suspension) were incubated with 1 mL of TMC-GA@PSS or TMC-GA/HP55@PSS solutions at concentrations ranging from 100 to 1000 μg/mL [44]. This mixture was incubated for two hours at 37 °C, followed by centrifugation at 3000 rpm for 10 min. Absorbance at 550 nm was measured using a Spark 10 M microplate reader (Tecan) after transferring 100 μL aliquots of each sample to a 96-well plate (NEST). Distilled water and normal saline served as the positive and negative controls, respectively.

Hemolysis ratio (%) = (A_sample_ − A_negative control_)/(A_negative control_ − A_negative control_) × 100% (4)

where A_sample_ was to the OD values of 4% RBC with samples, A_negative control_ was to the OD values of 4% RBC with normal saline, A _positive control_ was the OD value of 4% RBC with distilled water.

### 3.7. Cell Culture and Treatments

H9C2 cells, obtained from the American Type Culture Collection (ATCC), were seeded in 6-well plates at a density of 150,000 cells per well. Cultures were cultured in high-glucose DMEM supplemented with 10% fetal bovine serum (FBS) and 1% penicillin–streptomycin at 37°C under 5% CO_2_ in a humidified atmosphere for 24 h. Serum-free medium was then applied to replace the original medium and subjected to 12 h of starvation. Cardiac hypertrophy models were induced using different concentrations of Ang II, and drug treatment was carried out simultaneously: Control group, Ang II, Ang II + 10 μM telmisartan, Ang II + 100 μg/mL PSS, Ang II + 200 μg/mL PSS, Ang II + 400 μg/mL PSS. Cells were cultured for 24 h. Subsequently, the cell area was quantified, and the total cell proteins were extracted for further analysis.

#### 3.7.1. Real-Time Quantitative PCR

Total RNA was extracted from H9C2 cells with FreeZol Reagent (R711, Vazyme Biotech Co., Ltd., Nanjing, China), and the cDNA was synthesized with RNA PCR Kit (HiScript IV All-in-One Ultra RT SuperMix, Vazyme, China) with oligonucleotides (dT) as primers according to instructions. PCR amplification was quantified using ChamQ Universal SYBR qPCR Master Mix. The relative mRNA expression levels for cardiac hypertrophy-related genes were analyzed and normalized against the mRNA expression level of GAPDH. The primer sequences were listed in Table 1. 

#### 3.7.2. Cell Surface Area

The cell area was analyzed according to established methods [45]. In brief, cells were fixed using formaldehyde and permeabilized with 0.4% Triton X-100. Subsequently, cellular actin was stained in the dark using rhodamine-labeled phalloidin solution for 20 min, and finally, myocardial nuclei were stained in the dark using DAPI staining solution for 5 min. After three washes with PBS, the H9C2 cells were examined under the laser confocal microscope to assess changes in cell area.

#### 3.7.3. Western Blot Analysis

First, the protein samples were separated by SDS-PAGE, transferred to nitrocellulose membranes (Beyotime Biotechnology, Shanghai, China), and subsequently blocked in 5% BSA for 2 h at room temperature. Then, the samples were incubated at 4 °C for 16 h with specific primary antibodies: β-actin polyclonal antibody (#20536-1-AP, Proteintech Group, Inc., Wuhan, China) and NPPA Polyclonal antibody (#27426-1-AP, Proteintech Group, Inc., Wuhan, China). Subsequently, the blots were incubated with HRP-conjugated goat anti-rabbit secondary antibody (#SA00001-2, Proteintech Group, Inc., Wuhan, China) for 1 h at room temperature. Finally, the immunoreactive bands were detected using Omni-ECL™ Chemiluminescent Detection Kit (#SQ203, Epizyme Biomedical, Shanghai, China). Band intensities were quantified and normalized using the FiJi ImageJ software 13.0.6.

### 3.8. Animal Model and Treatment

Male C57BL/6J mice (9 weeks old, 22–25 g) were obtained from Jinan Pengyue Experimental Animal Breeding Co., Ltd., (Shandong, China). They were housed in a standard environment for 7 day of adaptive rearing and then underwent transaortic constriction (TAC) to establish a model of pressure overload–induced cardiac hypertrophy. All animal procedures were conducted in compliance with the National Research Council’s Guide for the Care and Use of Laboratory Animals and were approved by the Animal Ethics Committee of Ocean University of China (Approval No. OUC-SMP-2024-10-04). 

Forty-two mice were used in the experiment. Among them, thirty-six mice underwent TAC surgery and were randomized into six groups (*n* = 6): Model, Telmisartan (10 mg/kg/day), PSS (400 mg/kg/day), and TMC-GA/HP55@PSS at high, medium, and low doses groups (400, 200, and 100 mg/kg/day). The sham group (*n* = 6) only underwent surgery without ligation. The mice in all groups except the Sham group underwent TAC surgery. The drug was administered via gavage for four weeks. During this period, the sham and model groups were gavaged with an equal volume of saline daily. To evaluate the in vivo safety of nanoparticles, the body weights of mice in each group were measured, and their behaviors were observed. At the end of the treatment, the pharmacodynamic effects of PSS and its preparations on cardiac hypertrophy were assessed by echocardiographic measurements, cardiac index assay, and histopathological analysis.

#### 3.8.1. Echocardiography Analysis

After four weeks of treatment, echocardiographic measurements were performed using a Feyno small animal ultrasound system, as previously described [46]. The mice were anaesthetized with 1.5% isoflurane and positioned supinely. After the removal of chest hair, a mouse-specific probe was placed on the left hemithorax for M-mode cardiographic measurements according to the instruction manual. The main parameters recorded included left ventricular fractional shortening (LVFS), left ventricular ejection fraction (LVEF), left ventricular internal diameter during systole and diastole (LVIDs, LVIDd). 

#### 3.8.2. Histopathological Analysis

After 4 weeks of gavage treatment, the mice were weighed and executed, and their body weight (BW) was recorded. After the thoracic cavity was opened, the heart was rinsed with PBS, and the heart weight (HW) was recorded. At the same time, the length of the left tibia (TL) was accurately measured for each mouse. The ratio of the heart-to-body weight (HW/BW, mg/g) and heart weight to tibial length (HW/TL, mg/mm) was used as an indicator of cardiac hypertrophy [47].

Additionally, the hearts were excised and fixed using 4% paraformaldehyde, followed by paraffin embedding. The left ventricles were sectioned at 5 μm and subjected to wheat germ agglutinin (WGA) staining, Hematoxylin-Eosin (HE) staining, and Masson trichrome staining to assess left ventricular remodeling.

### 3.9. Statistical Analysis 

All values are presented as means ± standard deviation (SD). Data analysis was performed using GraphPad Prism 8.4.2 and Origin 2021 software. Statistical comparisons between data groups were made using one-way analysis of variance (ANOVA) followed by Bonferroni’s correction for post-hoc *t*-test with SPSS software (version 26.0). *p* < 0.05 was considered statistically significant, and *p* < 0.01 was considered highly significant.

## 4. Conclusions

In this study, a novel pH-responsive nanoparticle formulation (TMC-GA/HP55@PSS) was prepared to enhance the intestinal absorption of PSS and explore its potential in mitigating myocardial hypertrophy. The nanoparticles exhibited high encapsulation efficiency, stable performance under gastric conditions, and sustained release in the intestinal environment. Both in vitro and in vivo studies demonstrated that PSS attenuated cardiomyocyte hypertrophy and improved cardiac function in pressure overload-induced hypertrophic mice, with superior efficacy of TMC-GA/HP55@PSS. Importantly, our work indicated that combining bile acid transporter-mediated uptake with enteric protection was an effective dual strategy to improve the therapeutic outcomes of PSS. Beyond cardiac hypertrophy, this delivery platform offers a promising approach for the oral administration of other macromolecular polysaccharide drugs, and also provides *valuable* data support for its clinical application research.

## Figures and Tables

**Table 1:** Primer sequences used for qPCR assays.

Gene (Rat)	Forward Primer	Reverse Primer
ANP	CAGCACAATAGAGCCGCTGA	GGGCAGGAGCTTGAACACG
BNP	GCAGAAGCTGCTGGAGCTGA	ATCCGGAAGGCGCTGTCTTG
β-MHC	TTCGGGCGAGTCAAAGATGC	CCTTGTTCTCTGTTGCGTGC
GAPDH	ACCACAGTCCATGCCATCAC	TCCACCACCCTGTTGCTGTA

## Data Availability

Data presented in this study are available on request.

## Supplementary Materials

The following supporting information can be downloaded at https://www.mdpi.com/article/10.3390/md23090365/s1, Figure S1: Statistical analysis of body weight changes in mice; Figure S2: Representative images of HE staining of myocardial tissue. Scale bar: 100 μm; Figure S3: TEM image and particle size distribution of PSS nanoparticles. Scale bar: 500 nm.
