# Supplementary material for: Novel pH-Responsive PSS-Loaded Chitosan Matrix Nanoparticles Ameliorate Pressure Overload-Induced Cardiac Hypertrophy
Source: Mar Drugs. 2025 Sep 19;23(9):365. doi: 10.3390/md23090365 (PMC12471933; doi:10.3390/md23090365)
Supplement: Supplementary file 1 [file marinedrugs-23-00365-s001.zip › marinedrugs-3855095-supplementary.pdf]

Supporting Information

Novel pH-Responsive PSS-loaded Chitosan Matrix Nanoparticles Ameliorates Pressure Overload-induced Cardiac Hypertrophy

Meijie Xu [1,#], Zhen Fan [1,#], Dingfu Wang [1], Dan Li [1], Haimiao Zou [1], Yiting Xue [4], Shixin Wang [1,2,3,*], Chunxia Li [1,2,3,*]

1 Key Laboratory of Marine Drugs of Ministry of Education, Shandong Key Laboratory of Glycoscience and Glycotherapeutics, School of Medicine and Pharmacy, Ocean University of China, Qingdao, China; xumeijie@stu.ouc.edu.cn (M.X.); 270006039@qq.com (Z.F.); wangdingfu@stu.ouc.edu.cn (D.W.); ldan@stu.ouc.edu.cn (D.L.); haimiaozou@163.com (H.Z.);

2 Laboratory for Marine Drugs and Bioproducts, Qingdao Marine Science and Technology Center, Qingdao, China; shixin113@126.com (S.W.)

3 Laboratory of Marine Glycodrugs Research and Development, Marine Biomedical Research Institute of Qingdao, Qingdao, China; shixin113@126.com (S.W.)

4 School of Biology and Food Engineering, Suzhou University of Technology, Suzhou, China; xueyiting@cslg.edu.cn (Y.X.)

* Correspondence: lchunxia@ouc.edu.cn; shixin113@126.com

1. Materials and methods

1.1 *In vivo* safety evaluation of nanoparticles

To evaluate the in vivo safety of nanoparticles, the body weights of mice in each group were measured and their behaviors were observed.

1.2 Hematoxylin-Eosin (HE) staining of myocardial tissue

The isolated hearts were fixed with 4% paraformaldehyde for 24 h. The dehydrated tissues were embedded in paraffin and cut into 5 μm thick sections. Hematoxylin-Eosin (HE) staining was used to evaluate the myocardial structural changes[1].

1.3 Characterization of PSS nanoparticles

The morphology of TMC-GA@PSS and TMC-GA/HP55@PSS nanoparticles were observed by transmission electron microscopy (TEM, JEM-2100, Japan). The particle size distribution of the nanoparticles was statistically analyzed using ImageJ software.

2. Results and discussion

2.1 *In vivo* safety evaluation of nanoparticles

Over the 4-week period, the mice behaved normally and no significant change in body weight (**Fig. S1**), suggesting that the drug formulation is safe *in vivo*.

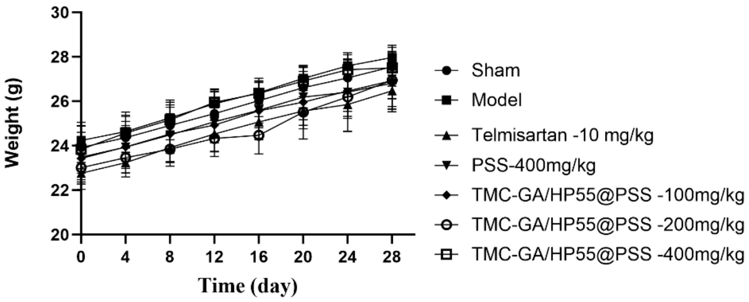

Fig. S1. Statistical analysis of body weight changes in mice.

2.2 Hematoxylin-Eosin (HE) staining of myocardial tissue

HE staining demonstrated that myocardial fiber arranged neatly with no obvious inflammatory cell infiltration (**Fig. S2**), suggesting the nanoparticles had good safety.

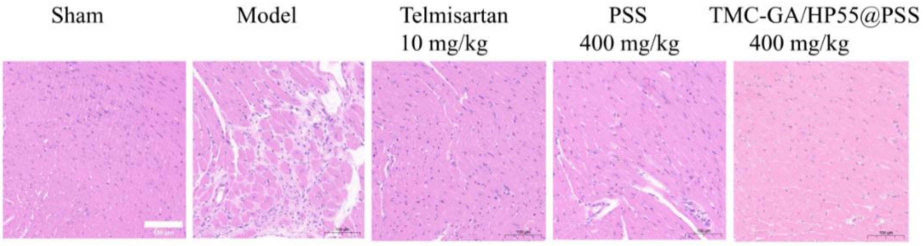

Fig. S2. Representative images of HE staining of myocardial tissue. Scale bar: 100 μm.

2.3 Characterization of PSS nanoparticles

As shown in **Fig.S3**, the morphology of TMC-GA@PSS and TMC-GA/HP55@PSS was spherical or spheroidal structure, observed by transmission electron microscopy, with the particle sizes 267.99 ± 59.48 nm and 337.56 ± 59.90 nm respectively.

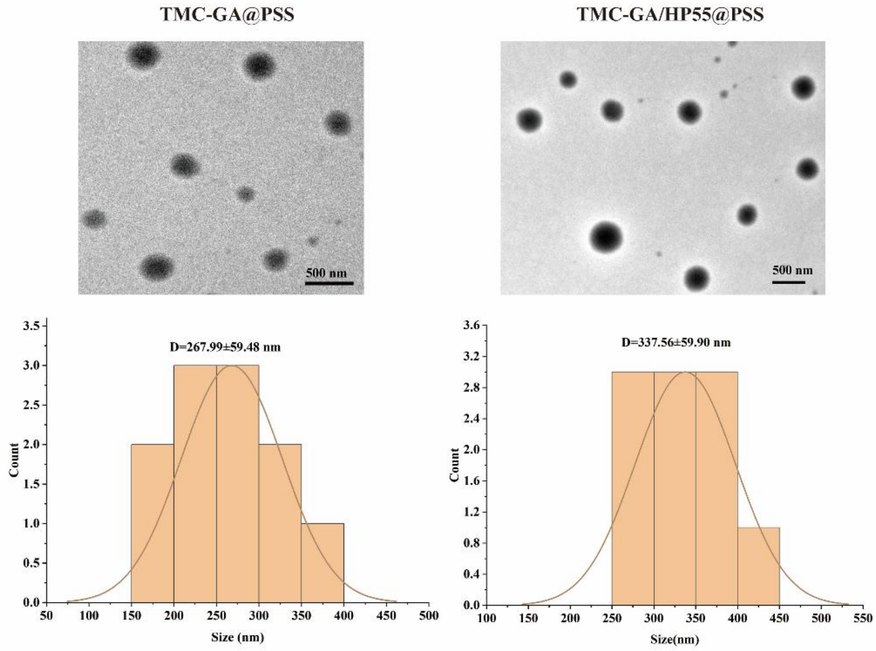

Fig. S3. TEM image and particle size distribution of PSS nanoparticles. Scale bar: 500 nm.

References

1. X. Qiu, S. Ma, D. Wang, Z. Fan, P. Qiu, S. Wang, C. Li, The development of multifunctional sulfated polyguluronic acid-based polymeric micelles for anticancer drug delivery, Carbohydr. Polym. 303 (2023) 120451. https://doi.org/10.1016/j.carbpol.2022.120451.
